# Revascularization of the graft in obliterative bronchiolitis after heterotopic tracheal transplantation

**DOI:** 10.14814/phy2.12690

**Published:** 2016-02-23

**Authors:** Simona Nemska, François Daubeuf, Nelly Frossard

**Affiliations:** ^1^ Laboratoire d'Innovation ThérapeutiqueUnité Mixte de Recherche 7200Centre National de la Recherche Scientifique‐Université de Strasbourg and Laboratoire d'Excellence MEDALISFaculté de PharmacieIllkirchFrance

**Keywords:** Lung transplantation

## Abstract

Obliterative bronchiolitis is the principal long‐term problem for lung transplant patients. One of the simplest and most reproducible animal models of obliterative bronchiolitis is heterotopic tracheal transplantation in subcutaneous tissue, where the graft is not primarily vascularized. We demonstrate here the rapid graft revascularization and the kinetics of expression of its angiogenic and lymphatic factors. We performed iso‐ and allotracheal transplantations harvested on day 0–21. The number of functional blood vessels, quantified after intravenous biotinylated dextran administration, increased from D0 (0 for both iso‐ and allografts) to D21 (44 ± 8 vessels/mm^2^ in isografts and 22 ± 3 in allografts, *P < *0.001 for both vs. D0). VEGF mRNA expression assessed by qPCR peaked on D1 (4.3‐fold increase in isografts and 4.0‐fold in allografts, *P < *0.0001 for both vs. D0), but receded thereafter. Angiopoietin‐1, involved in the maturation of the neoformed vessels, increased later on, by 6.2‐fold (*P < *0.05) in isografts and 11.5‐fold in allografts (*P < *0.001) by D21, and angiopoietin‐2 by 7.8‐fold in isografts (*P < *0.05) and 13.8‐fold in allografts (*P < *0.01). Although always present in the iso‐ and allografts, there were significantly more and larger LYVE1^+^ lymphatic vessels at D21 in allografts than in isografts. Thus, we demonstrate that tracheal grafts are rapidly revascularized by functional blood and lymphatic vessels, due to early VEGF and subsequent angiopoietins expression, which is a new advantage of this model, in addition to its ease of use, reproducibility, and viability in the absence of immunosuppressive treatment.

## Introduction

Lung transplantation (LT) is currently the only possible treatment for patients with end‐stage pulmonary diseases such as cystic fibrosis, COPD, or idiopathic pulmonary fibrosis. The main long‐lasting problem after LT remains the chronic lung allograft dysfunction (CLAD), including three phenotypes of persistent decline in pulmonary function: neutrophilic reversible allograft dysfunction (NRAD) which can be attenuated by azithromycin treatment; restrictive allograft syndrome (RAS), characterized by parenchymal infiltrates; and the bronchiolitis obliterans syndrome (BOS) with evidence of airway obliteration by inflammatory and fibroproliferative tissue observed in transbronchial biopsies, referred to as obliterative bronchiolitis (OB) (Suwara et al. 2014; Verleden et al. 2014). BOS occurs in around 50% of patients after the fifth year posttransplantation (Girgis et al., 1996; Hayes, 2011). It is a multifactorial process including primarily alloimmune T‐cell reactivity, human leukocyte antigen (HLA) antibody‐mediated rejection, autoimmune reactions, and environmental stress responses (Neuringer et al. 2005; Belperio et al. 2009; Grossman and Shilling 2009; Todd and Palmer 2011). No effective treatment for this postgraft complication is yet available.

Although the orthotopic lung transplantation in rodents seems to be the most relevant model for the study of OB in laboratory animals, it also shows clear disadvantages. Aside from its complexity to be performed in small animals, the allograft appears as edematous and necrotic when no immunosuppressive treatment is added in order to overcome the acute rejection (Yasufuku et al. 2002; Okazaki et al. 2007), decreasing the number of allografts developing OB lesions to 30% with gross variability (Zhang et al. 2008; De Vleeschauwer et al. 2012). Jungraithmayr et al. (2010) developed an immunosuppressive regimen with cyclosporine and rapamycin allowing the development of chronic rejection in 80% of the allografts in the rat, but the signs of fibroproliferation occurred only after 84 days posttransplantation and without airway obliteration. Considering the heterogeneity in the time period and in the obliterative signs, this model of chronic rejection after orthotopic lung transplantation in rodents seems of difficult use for proof‐of‐concept studies for novel OB treatments. More simple but reproducible models for first‐line proof‐of concept studies in OB may be used more efficiently. Such model is the heterotopic tracheal transplantation (HTT) in the subcutaneous tissue of an HLA‐mismatched recipient (Hertz et al. 1993). HTT is an easy‐to‐perform and reproducible model that develops characteristics of OB, that is, inflammatory cell infiltration and fibroproliferative graft obstruction, within 21 days. The process involves an early inflammatory phase with initial epithelial injury, followed by impaired epithelial regeneration and apoptosis in allografts as well as infiltration by alloreactive CD4^+^ and CD8^+^ T cells (Szeto et al. 2000; Neuringer et al. 2002; Richards et al. 2003, 2004). This inflammatory phase is followed by a fibroproliferative one, mediated by growth factors such as transforming growth factor *β*, platelet‐derived growth factor, and basic fibroblast growth factor; it leads to fibroblast and myofibroblast proliferation and collagen deposition and subsequently to tracheal obstruction (Al‐Dossari et al. 1995; Aris et al. 2002; Ramirez et al. 2004). Because the transplantation is in a heterotopic position, this model allows the study of the graft lesions over time, and avoids compromising the animal's life. As any animal model of a human disease, HTT has some disadvantages, most especially that the transplanted trachea is not functional, that is, in direct contact with air, and cannot be said to reflect the overall lung environment. In addition, the nonvascularization of the tracheal graft has been mentioned as its primary disadvantage (Grove et al. 2011; Neuringer et al. 2002; Oyaizu et al. 2003; Okada et al. 2004; Batirel et al. 2005; Maruyama et al. 2005; Zhou et al. 2005; McDyer 2007; Xu et al. 2008; Lau et al. 2009; Sato et al. 2009; Shah et al. 2000). A few studies have suggested that neovascularization of the graft might occur in HTT (Hertz et al. 1993; Nusair et al. 2005; McDyer 2007; Shah et al. 2000), but no histological evidence of the presence or functionality of blood vessels has been reported. Nor has lymphatic revascularization of the graft in this HTT model been shown, although lymphatic vasculature is necessary for the trafficking of lymphocytes and antigen‐presenting cells from the tissue to the lymph node that is essential for the immune response of graft rejection (Zawieja 2005; Choi et al. 2012).

We describe here the rapid revascularization of the graft by functional blood and lymphatic vessels after HTT, and provide histological evidence of this functional revascularization. Finally, we report the expression over time of angiogenic and lymphatic factors – VEGF (vascular endothelial growth factor), angiopoietin‐1 (Ang1) and ‐2, and podoplanin (Pdpn) – in the increasingly obliterated graft.

## Methods

### Animals

Animals were purchased from Janvier (Le Genest‐Saint‐Isle, France). Nine‐week‐old male Balb/cJ mice (H‐2K^d^) served as donors for iso‐ and allografts and as recipients for isografts. Nine‐week‐old male C57BL/6J mice (H‐2K^b^) were used as recipients for allografts. The animals were maintained on a 12/12‐h light/dark cycle, with food and water available ad libitum. All animals received humane care in compliance with the guidelines formulated by the French Ministry of Agriculture and of Higher Education and Research, and all procedures were performed in accordance with the European Community Council Directive of November 24, 1986 (86/609/EEC) and the French laws (agreement number 2015060415569684 APAFiS#768).

### Heterotopic tracheal transplantation

Donors were anesthetized with ketamine (154 mg/kg) (Imalgene, Merial) and xylazine (10 mg/kg) (Rompun, Bayer) in saline, administered intraperitoneally, and killed. Tracheas were procured after perfusion with EDTA 2.6 mmol/L in saline and immediately placed in ice‐cold PBS. Recipient mice were anesthetized by intraperitoneal administration of ketamine (51.4 mg/kg) and xylazine (3.3 mg/kg) in saline. Two subcutaneous pockets were dissected on the back of the mouse neck, and a half trachea from one donor was transplanted in one pocket and a half trachea from another donor in the other. Pockets were sutured with 5.0 silk suture (Silkam^®^). Mice were maintained for up to 21 days after transplantation. No immunosuppressant treatment was used. Transplanted tracheal grafts were harvested on day 0 (D0, at 1 h posttransplantation), D1, D3, D7, or D21, from mice deeply anesthetized by intraperitoneal injection of ketamine (154 mg/kg) and xylazine (10 mg/kg) in 0.1 mL saline, and perfused with EDTA 2.6 mmol/L in saline.

### Histology and immunohistochemistry

Harvested grafts (*n* = 6 per group) were fixed in paraformaldehyde 4% for 24 h, and paraffin‐embedded after successive lavages in ethanol (70–100%), followed by incubation in HistoClear (National Diagnostics, UK) and in paraffin at 60°C. Three levels (separated by 76 *μ*m) of 4 *μ*m transversal sections were cut per trachea and mounted on Superfrost glass slides (Fisher Scientific, France). After deparaffinization in xylene and successive rehydration, sections were treated with citrate buffer (10 mmol/L sodium citrate, 0.05% Tween 20) for antigen unmasking, and with endogenous peroxidase and alkaline phosphatase blocking reagent (Dako, S2003).

Tracheal sections were used for immunohistochemistry of the endothelial blood vessel CD31 marker (Goat polyclonal antibody, Santa Cruz, sc1506, 1:3000) and the lymphatic vessel LYVE1 (Lymphatic Vessel Endothelial Hyaluronan Receptor 1) marker (Rabbit polyclonal antibody, Abcam, ab14917, 1:1000), and for nuclei labeling with Neutral Red (Sigma, 72210, 1 g/L). Secondary antibodies were purchased from Jackson Laboratory and used at 1:500.

Vessel counts were performed (1) on tracheal tissue in the epithelial and subepithelial layer as well as around the cartilage for iso‐ and allografts; and (2) within the fibroproliferative tissue for allografts. Sections were observed on a Leica DM4000B microscope and analyzed with an Olympus DP72 camera with CellSensDimension software. The size of the CD31 vessels and of Lyve‐1 lymphatic vessels was measured with ImageJ software by their surface. Hematoxylin–eosin staining was performed on transversal tracheal sections from D0 to D21 in iso‐and allografts, and the epithelial loss was quantified (ImageJ).

### Dextran–biotin administration

On the day of graft harvest, the anesthetization took place 10 minutes after the intravenous administration in the tail vein of dextran‐biotin (Sigma, 14402; 80 mg/kg in 50 *μ*L). Four‐*μ*m tracheal sections (*n* = 6 per group) from dextran‐biotin‐treated animals were incubated directly with streptavidin peroxidase (Sigma, E2886, 1:500). Vessels were revealed with 3,3′‐diaminobenzidine, and nuclei were stained in a solution of Methyl Green (Sigma, 198080, 5 g/L, pH 4.2).

### RNA extraction, RT‐PCR, and qPCR

For mRNA quantification by RT‐qPCR in tracheas harvested at D0, D1, D3, D7, and D21, total RNA was extracted from pools of four half‐tracheas (*n* = 6 pools per group) with the RNAeasy Micro Kit (Qiagen) and quantified on a Nanodrop^®^. One *μ*g of RNA was reverse‐transcribed with mMLV reverse transcriptase (Promega). cDNA was used for qPCR, with the Applied Biosystem primers for VEGF isoform A (Mm01281449_m1), HIF1*α* (Mm00468869_m1), angiopoietin‐1 (Mm00456503_m1), angiopietin‐2 (Mm00545822_m1), podoplanin (Mm00494716_m1), and HPRT1 (Mm01545399_m1) as a housekeeping gene. An ABI Prism 7000 thermocycler performed 40 cycles of qPCR for each gene. Expression of the mRNA of the gene of interest was referenced to HPRT1 (hypoxanthine phosphoribosyltransferase 1) expression (2^−ΔCt^) and calibrated to the D0 values (2^−ΔΔCt^).

### Statistical analysis

Data are expressed as means ± SEM. Statistical analyses were performed with one‐way ANOVA and then Bonferroni's post hoc test for comparison of selected data pairs. Differences were considered significant when *P < *0.05.

## Results

### Vascularization of tracheal and fibroproliferative tissues after heterotopic tracheal transplantation

**Figure 1:**
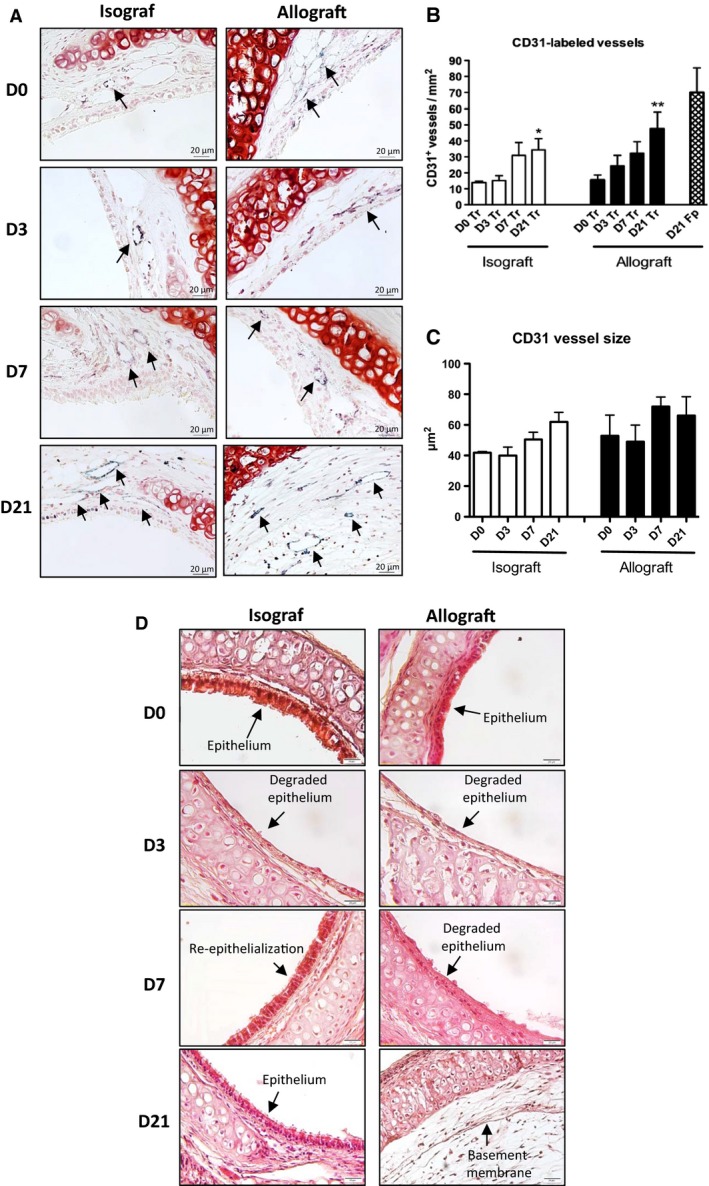
Vascularization of iso‐ and allografts after heterotopic tracheal transplantation. Paraffin‐embedded sections of the tracheal grafts were immunostained for the endothelial marker CD31. (A) CD31^+^ vessels were visualized in the tracheal tissue (D0–D21, iso‐ and allograft) and in the fibroproliferative tissue obstructing the tracheal lumen (D21, allograft) after streptavidin‐peroxidase revelation and stained blue with Histogreen® (dark arrows). Cell nuclei are colored red after Neutral Red staining. Gx400. (B) Counts of CD31^+^ vessels per mm^2^ in the tracheal tissue (Tr; Black and white blocks). The count of CD31^+^ vessels per mm^2^ in the fibroproliferative tissue (Fp; Pattern block) is presented for the D21 allograft. Three levels of sections were counted for each trachea. Data represent mean values (blocks) ± SEM (bars) (*n* = 6). *P < 0.05, **P < 0.01 compared to D0. (C) The CD31 vessel size was quantified by ImageJ software and represented as mm^2^ surface from D0 to D21 in both iso‐ and allografts. (D) Kinetics of the epithelial regeneration in isografts and of the epithelial loss in allografts. Hematoxylin and eosin staining of transversal cuts of tracheas iso‐ (left) and allografts (right) at D0, D3 D7 and D21 after transplantation.

Immunolabeling of tracheal sections for the endothelial marker CD31 showed CD31^+^ endothelium both in iso‐ and allografts. As Figure 1A shows, CD31^+^ vessels were located in the subepithelial layer of tracheas and in the tissue surrounding the cartilaginous rings. From D0 to D21 after transplantation, the number of CD31^+^ blood vessels increased progressively in tracheal tissue in allografts (Fig. 1B), from 14 ± 1 at D0 to 48 ± 10 vessels/mm^2^ at D21 (*P < *0.01 vs. D0), and in isografts, from 15 ± 4 at D0 to 34 ± 9 vessels/mm^2^ at D21 (*P < *0.05 vs. D0) (Fig. 1B). This number did not differ significantly between these graft types. We also observed CD31^+^ blood vessels within the fibroproliferative tissue, obstructing the tracheal lumen in allografts on D21 (70 ± 15 vessels/mm^2^) (Fig. 1B). We analyzed the size of the CD31 vessels in iso‐ and allografts from D0 to D21, but we did not find any significant modification of the vessel size (Fig. 1C). CD31^+^ vessels are of size between 10 *μ*m^2^ and 120 *μ*m^2^.

The rate of epithelial loss was quantified in iso‐ and allografts at D0, D3, D7, and D21 posttransplantation on hematoxylin–eosin stained sections to characterize and validate the OB model (Fig. 1D). In isografts, the tracheal epithelium is degraded (44 ± 12% of epithelial loss) at D3, and further re‐epithelialized at D7 (only 5 ± 2% of epithelial loss) and D21 (0 %). In allografts by contrast, the epithelium is degraded by D3 (83 ± 7%), and remains further degraded at D7 (67 ± 8%) and D21 (100%) (Table 1).

**Table 1:** Quantification of the percentage of degraded epithelium in iso‐ and allografts at D0, D3, D7 and D21 (Mean ± SD)

% degraded epithelium	D0	D3	D7	D21
Isograft	0	44 ± 12	5 ± 2	2 ± 1
Allograft	0	83 ± 7	67 ± 8	100

### Functionality of blood vessels vascularizing the graft

**Figure 2:**
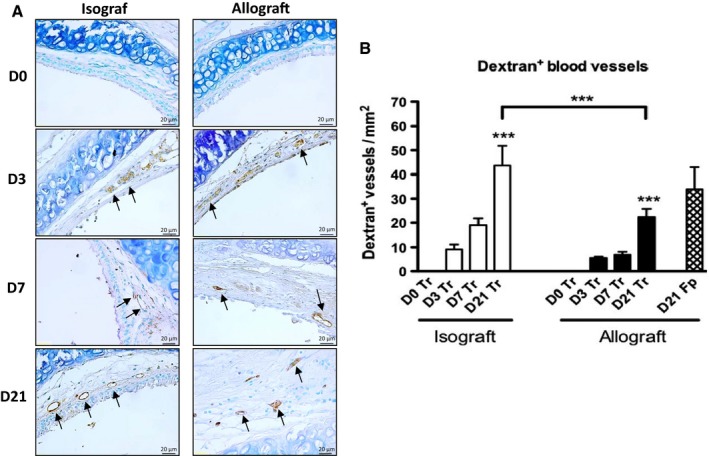
Functional vascularization of iso‐and allografts after heterotopic tracheal transplantation. Biotinylated dextran (80 mg/kg) was administered IV before tracheal harvest. (A) Blood vessels were visualized in the tracheal tissue (D0–D21, iso‐ and allograft) and in the fibroproliferative tissue obstructing the tracheal lumen (D21, allograft) after streptavidin‐peroxidase incubation of the paraffin‐embedded sections and were then stained brown with 3,3′‐Diaminobenzidine (dark arrows). Cell nuclei are colored blue after Methyl Green staining. Gx400. (B) Count of dextran^+^ vessels per mm^2^ in the tracheal tissue (Tr; Black and white blocks). The count of Dextran^+^ vessels per mm^2^ in the fibroproliferative tissue (Fp; Pattern block) is presented for the D21 allograft. Three levels of sections were counted for each trachea. Data represent mean values (blocks) ± SEM (bars) (*n* = 6). ***P < 0.001 compared to D0.

To demonstrate the functionality of the blood vessels, we labeled them in vivo with dextran, which binds to blood vessel endothelium. Dextran‐labeled blood vessels were shown in the subepithelial layer and in the tissue surrounding cartilaginous rings in both iso‐ and allografts (Fig. 2A). Figure 2B presents the labeled vessel counts at each time point. As expected, there were no functional blood vessels on D0 in either graft type. The number increased progressively from D3 to D21: 9 ± 2 vessels on D3 to 44 ± 8 on D21 (*P < *0.001 vs. D0) in isografts and 5 ± 1 on D3 to 22 ± 3 on D21 (*P < *0.01 vs. D0) in allografts. These increases suggest that an angiogenic process occurs after transplantation. The number of dextran‐labeled vessels at D21 also differed significantly between isografts and allografts (*P < *0.001) (Fig. 2B). Additionally, we observed dextran‐labeled vessels within the fibroproliferative tissue of allografts (34 ± 9 vessels/mm^2^), indicating that the OB model is vascularized with functional blood vessels (Fig. 2B).

### Expression of angiogenic factors in the graft

To understand the molecular mechanisms underlying our histological observation of the increased vascularization in the grafts posttransplantation, we analyzed mRNA expression of the angiogenic factors VEGF, angiopoietin‐1 (Ang1), and ‐2 (Ang2).

**Figure 3:**
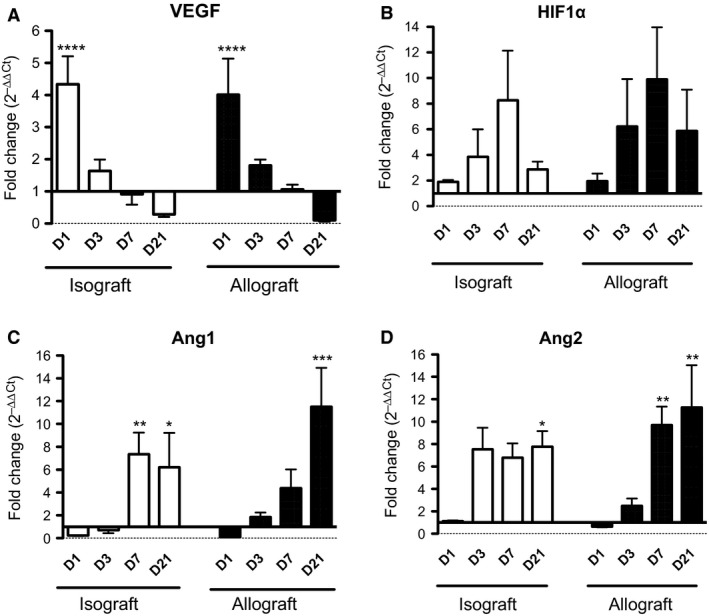
Relative expression of mRNA of angiogenic genes in tracheal grafts. qPCR was performed with the Applied Biosystem Gene Expression Assay in pools of four half‐tracheas for each time point (*n* = 6 pools). The results are calculated by the ΔΔCt method (ΔΔCt = ΔCt sample − ΔCt calibrator, where ΔCt is the mean value at D0 iso‐ or allograft). (A) Quantification of the expression of VEGF mRNA in the grafts. ****P < 0.0001 compared to D0. No significant difference of the D0 levels of VEGF mRNA expression (2^−ΔCt^ values) between isografts (6.3 ± 0.7) and allografts (8.8 ± 1.6). (B) Quantification of the expression of HIF1*α* mRNA in the grafts. No significant difference of the D0 levels of HIF1*α* mRNA expression (2^−ΔCt^ values) between isografts (2.2 ± 1.1) and allografts (1.5 ± 0.5). (C) Quantification of the expression of angiopoietin‐1 mRNA in the grafts. ***P < 0.001, **P < 0.01, *P < 0.05 compared to D0. No significant difference of the D0 levels of Ang1 mRNA expression (2^−ΔCt^ values) between isografts (1.3 ± 0.3) and allografts (2.2 ± 1.0). (D) Quantification of the expression of angiopoietin‐2 mRNA in the grafts. **P < 0.01, *P < 0.05 compared to D0. No significant difference of the D0 levels of Ang2 mRNA expression (2^−ΔCt^ values) between isografts (2.1 ± 1) and allografts (2.6 ± 1.5).

VEGF is an angiogenic factor that promotes angiogenesis and neovascularization in tissue. VEGF mRNA expression peaked at D1 in iso‐ (4.3‐fold increase vs. D0, *P < *0.0001) and allografts (4.0‐fold increase vs. D0, *P < *0.001) (difference between iso‐ and allografts not significant) (Fig. 3A). It then decreased progressively (D3, D7), with levels lower on D21 than on D0 (Fig. 3A). As VEGF expression is reported as modulated by hypoxia, we also analyzed the expression of the hypoxia transcription factor HIF1*α*, but did not observe any increase on D1 when hypoxia is occurring (Fig. 3B). Interestingly, HIF1*α* shows a trend, although nonsignificant, to increase on D7, when the vascularization is re‐established both in iso‐ and allografts (Fig. 3B).

Ang1 is an angiogenic marker that plays an important role in blood vessel remodeling and maturation and thereby promotes blood vessel stability. We observed increased mRNA expression of Ang1 in iso‐ and allografts from D7 posttransplantation, by 7.4‐fold in isografts compared to D0 (*P < *0.01) and 4.4‐fold in allografts (NS vs. D0) (Fig. 3C); at D21, these fold‐increases were 6.2 in isografts (*P < *0.05) and 11.5 in allografts (*P < *0.001), compared to D0 (Fig. 3C). This high expression of Ang1 at a stage when VEGF has fallen below baseline values suggests that Ang1 might be important in the OB model for stabilization of the blood vessels newly formed under the primary action of VEGF. Ang2 mRNA expression increased significantly in allografts from D7 to D21: 9.7‐fold at D7 (*P < *0.05 vs. D0) and 11.3‐fold at D21 (*P < *0.01 vs. D0) (Fig. 3D). Here again, this late expression in allografts suggests that Ang2 has a role in the vascular remodeling associated with the allogeneic fibroproliferation. In isografts, the levels of mRNA expression remained rather stable from D3 to D21: a 7.5‐fold increase at D3 (NS vs. D0) versus 7.8‐fold at D21 (*P < *0.05 vs. D0).

### Lymphatic vessels vascularize the OB model

**Figure 4:**
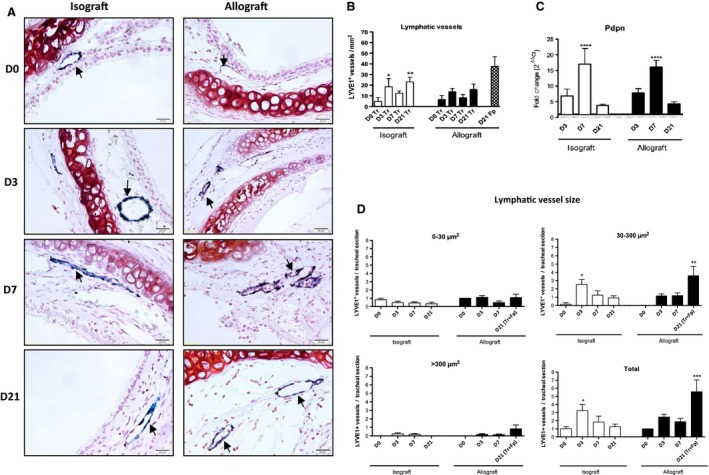
Lymphatic vascularization after heterotopic tracheal transplantation. Paraffin‐embedded sections of the tracheal grafts were immunostained for the lymphatic marker LYVE1. (A) LYVE1^+^ vessels are visualized in the tracheal tissue (D0–D21, iso‐ and allograft), and in the fibroproliferative tissue obstructing the tracheal lumen (D21, allograft) after streptavidin‐peroxidase revelation; they are stained blue with Histogreen^®^ (dark arrows). Cell nuclei are colored red after Neutral Red staining. Gx400. (B) Count of LYVE1^+^ vessels per mm^2^ in the tracheal tissue (Tr; Black and white blocks). The count of LYVE1^+^ vessels per mm^2^ in the fibroproliferative tissue (Fp; Pattern block) is represented for the D21 allograft. Three levels of sections were counted for each trachea. Data represent mean values (blocks) ± SEM (bars) (*n* = 6). **P < 0.01, *P < 0.05 compared to D0. (C) Expression of the lymphatic marker podoplanin in the graft after heterotopic tracheal transplantation. qPCR was performed with the Applied Biosystem Gene Expression Assay in pools of four half‐tracheas for each time point (*n* = 6 pools). Results are calculated by the ΔΔCt method (ΔΔCt = ΔCt sample − ΔCt calibrator, where ΔCt is the mean value on D0 for the iso‐ or allograft). *P < 0.05, ****P < 0.0001 compared to D0. (D) Measurement of the surface area of the lymphatic vessels. The surface area (*μ*m^2^) of lymphatic vessels LYVE1^+^ in the entire tracheas was measured with ImageJ software. The figure presents the mean value of the number of lymphatic LYVE1^+^ vessels/section ±SEM (bars), counted for each time point for iso‐ and allografts. Vessels are grouped as three sizes: 0–30, 30–300, >300 *μ*m^2^ are shown. Three levels of sections were counted for each trachea. *P < 0.05, **P < 0.01, ***P < 0.001 compared to D0.

Immunostaining for the lymphatic vessel marker LYVE1 (Fig. 4A) showed LYVE1‐labeled vessels in tracheal tissue in the subepithelial layer of both iso‐ and allografts, as well as within the fibroproliferative tissue in allografts at D21. Their number increased in tracheal tissue (Tr) from 5 ± 4 at D0 to 23 ± 4 vessels/mm^2^ at D21 in isografts (*P < *0.01) and from 7 ± 4 at D0 to 16 ± 5 at D21 in allografts (NS) (Fig. 4B). In addition, lymphatic vessels were observed in the fibroproliferative tissue (Fp) obstructing the allografts (38 ± 9 vessels/mm^2^) and are represented as a separate block (D21 Fp) on Figure 4B. Lymphatic vessel size was analyzed in iso‐ and allografts in the tracheal and fibroproliferative tissue in the whole tracheal sections in each group (D0, D3, D7, D21). Vessels of 0–30, 30–300, and >300 *μ*m^2^ were counted (Fig. 4D). Vessels of 30–300 *μ*m^2^ were observed in isografts, mostly at D3 (2.5 ± 0.6 vessels/section, *P < *0.05 vs. D0), whereas in allografts any significant increase occurred until D21 (3.6 ± 1.1 vessels/section, *P < *0.01 vs. D0) (Fig. 4C). The largest vessels (>300 *μ*m^2^) were totally absent at D21 from isografts, but there were a few of these very large vessels at D21 in allografts (0.8 ± 0.4 vessels/section) (Fig. 4D). For lymphatic vessels as a whole (Total on Fig. 4D), a significant increase in the total number of lymphatic vessels – including tracheal and fibroproliferative tissue – occurred late in allografts, on D21 (5.6 ± 1.4 vessels/section, *P < *0.001 vs. D0), at the same time that lymphatic vessels developed in the fibroproliferative tissue (Fig. 4B). In the isografts, in contrast, the total number of lymphatic vessels increased early, on D3 (3.2 ± 0.8 vessels/section, *P < *0.05) and decreased thereafter to values close to those of controls (Fig. 4D).

We also quantified the expression of podoplanin (Pdpn), a mucin‐type transmembrane glycoprotein specifically expressed by lymphatic endothelial cells. mRNA expression peaked on D7, similarly in iso‐ and allografts (17‐ and 16‐fold increase vs. D0, respectively, *P < *0.001) (Fig. 4C). No significant difference was found between the D0 levels of Pdpn mRNA expression (2^−ΔCt^ values) in isografts (2.2 ± 1.2) and allografts (0.8 ± 0.2).

## Discussion

The murine heterotopic tracheal allotransplantation HTT model is a common model for studying obliterative bronchiolitis or OB development after transplantation. The primary advantages include that it allows the detailed study of the mechanisms of inflammation and fibroproliferation associated with the progressive obstruction of the airways, as in this heterotopic position it is no longer life threatening. In human lung transplantation, because of the direct contact of the transplanted lung with the air and along with the classical immunosuppressive posttransplantation treatment, patients are often subjects to viral and bacterial infections. Neutrophils are in this case infiltrating the lung and we observe the so‐called NRAD form of CLAD (discussed in the Introduction), which cannot be modeled by the classic HTT. Other signal present in humans and which is cause for OB is the gastro‐esophageal reflux. Although we state that HTT is a good and not aggressive model for first proof‐of‐concept studies of OB treatment, and we bring here a new advantage, which is the revascularization of the graft. The nonvascularization of the tracheal graft has been mentioned earlier as a primary disadvantage of the model (Grove et al. 2011; Neuringer et al. 2002; Oyaizu et al. 2003; Okada et al. 2004; Batirel et al. 2005; Maruyama et al. 2005; Zhou et al. 2005; McDyer 2007; Xu et al. 2008; Lau et al. 2009; Sato et al. 2009; Shah et al. 2000). However, processes occurring after HTT, such as the infiltration of inflammatory cells into the allograft and the re‐epithelialization of the isograft, nonetheless suggest potent functional graft revascularization. A few reports provide evidence that the graft is revascularized (Belperio et al. 2005; Fan et al. 2013), but no one has ascertained the functional blood and lymphatic vasculature in the graft and in the obliterative trachea. This was the aim of our study.

To do so, immunostaining for the marker of blood endothelial cells CD31 (Zhao et al. 2010) shows blood vessels in the grafted trachea, located within the subepithelial layer and around the cartilaginous rings. We report a significant increase in the number of CD31^+^ vessels from D0 to D21 in both iso‐ and allografts, suggesting occurrence of posttransplantation angiogenesis. This is in contrast to the report by Zhao et al. (2010) of CD31‐immunostaining in allografts (Balb/c to C57BL/6), with no increase in terms of densitometric units between D3 and D21, whereas we clearly quantified an objective increased number of CD31‐labeled blood vessels in the grafted tracheal tissue. Another study (Batirel et al. 2005) reported, consistently with our results, no difference in the number of CD31^+^ vessels from D14 to D28 in iso‐ and allografts but included no comparison to an earlier time point posttransplantation. Another important finding of our study is the presence of CD31^+^ vessels within the obstructive fibroproliferative tissue in allografts at D21, that is, in the OB model. This indicates that the fibroproliferative tissue is highly vascularized, and suggests that fibrosis and vascularization occur simultaneously. These results obtained in murine heterotopic tracheal allografts are consistent with data from lung transplant patients with OB reporting that the number of CD31‐labeled vessels in small airways increases with airway obliteration (Luckraz et al. 2004).

We then sought to demonstrate the functionality of these CD31^+^ vessels through their in vivo labeling by dextran‐biotin, which binds to the lectin structures of the vascular endothelium (Gonzalez‐Castillo et al. 2003), and allows to follow the functional vascularization of the graft over time. As expected, our results show no functional blood vessels at D0 (i.e., at 1 h posttransplantation) in iso‐ and allografts, as no primary vasculature was reconnected at transplantation. We then show the functional revascularization of the tracheal tissue as early as D3, which increases over time up to D21 both in iso‐ and allografts. In accordance with the CD31 labeling, the functional dextran‐labeled vessels were mainly located within the subepithelium both in iso‐ and allografts. The number of dextran‐labeled vessels was twice as high in isografts as in allografts on D21, suggesting the allogeneicity may affect posttransplant angiogenesis and vascular growth. This is the first demonstration to our knowledge of the kinetics of functional blood vessels in the tracheal graft.

In addition, we show in isografts that functional revascularization occurs progressively in parallel with epithelial regeneration, which is almost complete by D21 (Neuringer et al. 2002; Lau et al. 2009). We confirm in allografts that the alloreaction prevents epithelial regeneration, as epithelium is still degraded on D7; rather than epithelial regeneration, inflammatory cell infiltration occurs, followed by fibroproliferation, which is nearly complete by D21 (Hertz et al. 1993; Neuringer et al. 2002; KleinJan et al. 2008; Lau et al. 2009). We are the first to demonstrate, however, that OB in allografts is associated with fewer dextran‐labeled functional blood vessels in the tracheal tissue compared with isografts.

We next investigated the expression of the angiogenic factors VEGF, Ang1, and Ang2, during revascularization of the graft and vascular remodeling in the allografts. VEGF acts on the maintenance of normal vascularity in the lung (Voelkel et al. 2006). The peaking of VEGF expression on D1 as compared to D0 both in iso‐ and allografts suggests rapid signaling for angiogenesis after transplantation. VEGF decreased thereafter, from D3 to D21. We measured in parallel the expression of the hypoxia‐inducible transcription factor HIF1*α* controlling the VEGF gene (Forsythe et al. 1996). HIF1*α* expression was not modified at D1 when VEGF peaked, in contrast to the reported increase in HIF1*α* protein expression in a mouse model of orthotopic tracheal allotransplantation (Jiang et al. 2011). This does not exclude, however, the possibility of stabilization of the HIF1*α* subunit and subsequent activation of the expression of VEGF gene in our model (Pages and Pouyssegur 2005; Semenza et al. 2000; Mura et al. 2004; Kugathasan et al. 2005).

The angiopoietins (Ang1 and Ang2) are other important angiogenic factors. Their expression significantly increased later in our model, on D21 in iso‐ and allografts compared to D0, that is, after VEGF expression has ended. Ang1 and Ang2 have highly similar sequences and are both ligands of the Tie2 receptor (*tunica intima endothelial tyrosine kinase 2*), expressed by vascular endothelial cells (Fiedler et al. 2003; Brindle et al. 2006; Kumpers et al. 2010), but distinct functional and cell‐signaling characteristics. Ang1 is reported to play a role in the maturation and stabilization of newly formed blood vessels through activation of Tie2 autophosphorylation and intracellular signaling; it induces endothelial cell migration, sprouting, and tube formation (Koblizek et al. 1998; Witzenbichler et al. 1998; Hayes et al. 1999; Jones et al. 1999; Kwak et al. 1999). A late increased expression of Ang1 is observed in our study both in iso‐ and allografts. We propose this allows maturation of the blood vessels initially newly formed under the influence of VEGF, stabilizing their structural integrity (Augustin et al. 2009). This is consistent with reports of the chemotactic properties of Ang1 (Witzenbichler et al. 1998; Metheny‐Barlow et al. 2004), that might play a role in the increased Ang1 expression we observed in allografts, significantly higher than in isografts at D21.

In contrast, Ang2 prevents Ang1 binding to Tie2, thereby inhibiting Tie2 autophosphorylation, and functions as a natural Tie2 antagonist to regulate vascular development (Maisonpierre et al. 1997). Hence, Ang2 is required for normal vascular remodeling (Gale et al. 2002) and vessel regression, especially in the absence of VEGF (Holash et al. 1999; Lobov et al. 2002; Brindle et al. 2006; Augustin et al. 2009). Ang2 was also expressed late as compared to VEGF in the grafts. In isografts, Ang2 expression appears fairly stable from D3 to D21, whereas it increases progressively in allografts from D7 to D21. This is also consistent with aberrant angiogenesis reported in lung biopsies from BOS patients (Belperio et al. 2005). This may be related to Ang2's role in vascular remodeling and angiogenesis regression, as reported in lung tumors where blocking Ang2 improves the quality of junctions between endothelial cells (Holopainen et al. 2012). Thus, we hypothesize that allogeneicity helps to promote the expression of Ang2 in allografts, that leads to vascular remodeling, and thus slows the vascularization process. Endothelial and bronchial epithelial cells are angiopoietins producing cells (Mura et al. 2004). In isografts which are re‐epithelialized at D7 and 21, Ang1 and 2 may be expressed by endothelial and epithelial cells in the tracheal graft; in allografts where the epithelium is degraded, the endothelial cells of the developing vessels might be the source of angiopoietins, in particular in the fibroproliferative tissue in allografts. This obviously suggests a complex balance between vessel maturation and regression, supported by the smaller number of functional vessels in the tracheal tissue of allografts compared to isografts.

In addition to blood vessel revascularization, we report here that LYVE1^+^ lymphatic vessels (endothelial hyaluronan receptor) are located within the subepithelium of the tracheal tissue and around the cartilage, as well as within the fibroproliferative tissue of the allografts. The larger lymphatic vessels (30–300 and >300 *μ*m^2^) occur more often in allografts in all these locations; indeed, the largest vessels (>300 *μ*m^2^) cannot even be found in isografts at D21. This is consistent with findings from the HTT in the omentum, where lymphatic vasculature has been reported in allograft fibroproliferative tissue (Tikkanen et al. 2006). Lymphatic vessels are involved in the trafficking of antigen‐presenting cells to the graft. We hypothesize that the lymphatic supply is involved in the re‐epithelialization in isografts (D3) (Hegab et al. 2012), and required for the alloreaction in allografts (D21).

We also show that expression of the mucin‐type transmembrane glycoprotein podoplanin increases and peaks at D7 in both iso‐ and allografts. Its precise function is not yet well understood. It is expressed by the lymphatic endothelial cells during lymphangiogenesis, induces platelet aggregation, which blocks lymphaticovenous connections, and helps separate the blood and lymphatic vascular systems (Suzuki‐Inoue et al. 2006; Uhrin et al. 2010). Pdpn is also required for maintaining the segregation of blood and lymphatic vessels during tissue remodeling (Fu et al. 2008). In our experiments, however, Pdpn expression did not differ between iso‐ versus allografts at D21. This finding suggests that it may be involved in lymphatic vessel maintenance in the grafts, but not in the size of the lymphatic vessels. Further investigation of the lymphatic vasculature in tracheal grafts would be useful for understanding its role in the pathophysiological processes of OB. Nevertheless, we show for the first time the presence of lymphatic vessels in tracheal grafts after HTT in the subcutaneous tissue of mice.

## Conclusion

This study is the first to clearly demonstrate the vascularization of iso‐ and allografts and to quantify it by counting the functional blood vessels and lymphatic vessels after subcutaneous murine HTT. The angiogenic factors VEGF, Ang1, Ang2, and podoplanin mediate the formation of the vascular and lymphatic network in the transplanted tracheas as OB progresses. Our data thus provide new information for our understanding of HTT as a model for OB. This study also sheds light on a new advantage of this model, in addition to its ease of use, reproducibility, and viability in the absence of immunosuppressive treatment, as a valuable model for performing proof‐of‐concept studies in OB development during chronic lung allograft dysfunction.

## Conflict of Interest

The authors have no conflicts of interest to disclose. The funders had no role in study design, data collection and analysis, decision to publish, or preparation of the manuscript.
